# Preoperative platelet-to-lymphocyte ratio and neutrophil-to-lymphocyte ratio as predictors of clinical outcome in patients with gallbladder cancer

**DOI:** 10.1038/s41598-018-38396-4

**Published:** 2019-02-12

**Authors:** Sha Zhu, Jing Yang, Xiwei Cui, Yunuo Zhao, Zhihang Tao, Fan Xia, Linyan Chen, Juan Huang, Xuelei Ma

**Affiliations:** 1 State Key Laboratory of Biotherapy and Cancer Center, West China Hospital, Sichuan University, and Collaborative Innovation Center for Biotherapy, Chengdu, China; 2 West China School of Medicine, West China Hospital, Sichuan University, Chengdu, 610041 P. R. China; 3 Department of Hematology, Sichuan Academy of Medical Sciences, Sichuan Provincial People’s Hospital, School of Medicine, University of Electronic Science and Technology of China, Chengdu, 610031 Sichuan China

## Abstract

Some inflammatory biomarkers are associated with the post-surgical prognosis in cancer patients. However, their clinical importance in gallbladder cancer has rarely been explored. The aim of this study is to assess the efficacy of surgical intervention and the effectiveness of preoperative test on neutrophil-to-lymphocyte ratio (NLR), platelet-to-lymphocyte ratio (PLR) and monocyte-to-lymphocyte ratio (MLR) for predicting the prognosis in gallbladder cancer patients. In this study, a total of 255 gallbladder cancer patients were retrospectively selected. For each patient, we recorded his/her treatment algorithm (with or without surgery) and their preoperative inflammatory biomarkers, as well as their detailed survival information for 5 years. A total of 216 patients received surgical intervention and the other 39 chose conservative treatment. The median survival time was 4.6 months for non-surgical group (P < 0.001), and 12.2 months for surgical intervention group. Among the surgical group, ROC analysis showed the AUC of NLR, PLR and MLR were 0.675 (95% CI: 0.600 to 0.751, P < 0.001), 0.599 (95% CI: 0.520 to 0.677, P = 0.017) and 0.607 (95% CI: 0.529 to 0.686, P = 0.009), respectively. In conclusion, surgical intervention did improve the overall survival, and elevated NLR and MLR before surgery are associated with shorter OS of GBC patients.

## Introduction

Gallbladder cancer (GBC) is the most common kind of malignant tumors originated from the biliary tract and is also a relatively uncommon disease compared to other types of cancer^1^. GBC is marked by its insidious onset. Often it is after the incipient symptoms have started to occur when the tumor tissue is discovered inadvertently. At that time, patients are likely to present a rather poor recovery outcome, with a 5-year survival rate less than 5%^2^. Broadly speaking, GBC has a rather poor prognosis, being “the most common and aggressive malignancy of the biliary tree”^1,3^. Hence, the treatment of GBC remains a significant challenge in modern medical care.

Although complete surgical resection is considered the only potentially curative treatment^4^, the surgery is often very challenging as the gallbladder is next to numerous vital structures anatomically. Since these interventions are pretty radical procedures, some hold the opinion that other treatment methods such as traditional Chinese medicine or immuno-biological cancer therapy might be of more importance^5^. To the best of our knowledge, we find that data on the actual prognosis of surgical intervention in patients with gallbladder cancer is still scarce.

Furthermore, from some previous studies into the mechanisms and kinetics of malignancies, we now know that many of the malignancies are initiated by chronic infection, which accounts for nearly 15% malignancies worldwide^6^. Chronic inflammation arising from it may have crucial influence on tumorigenesis. We also learn that plenty of inflammatory cells as well as innate immune system signaling molecules are of great significance in the occurrence and development of many kinds of cancers, including GBC. The neutrophil/lymphocyte ratio (NLR), monocyte/lymphocyte ratio (MLR) and blood platelet/lymphocyte ratio (PLR) are all biomarkers of host inflammation, and they can be considered as prognostic factors in several cancers, as their elevation are associated with poor overall survival^7–9^. Therefore, we naturally went to pursue the exact correlation between these biomarkers and the prognosis of GBC.

GBC has a heterogeneity and insidious onset in clinical presentation, combined with poor prognosis as well as subsequently complex clinical management of this disease, therefore, it is imperative for us to raise some more prognosis biomarkers in order to provide GBC patients with more personalized and effective treatment, hopefully. Thus, the first objective of the study is to analyze outcomes of surgical interventions in previous GBC patients in our hospital to determine that whether surgical intervention can prolong patients’ life span. The second goal is to analyze their preoperative NLR, PLR and MLR results to find out if there is a correlation which can be used into predicting in the future, as well as finding out which kind of patients may benefit most in this procedure.

## Results

### Patient Characteristics

A total of 255 patients were diagnosed with GBC at our institution between 2009 and 2017 and which formed our study group.

Among these cases: 155 (60.8%) females, 100 (39.2%) males. Median age was 63 years (range 33–96). This cohort was clinically heterogeneous in terms of tumor stage, it consisted of 17 (6.7%) stage I GBC, 33 (12.9%) stage II GBC, 82 (32.2%) stage III GBC, 123 (48.2%) stage IV GBC, respectively (Table 1). The pathological findings of resected specimens presented that 117 (45.9%) of the patients had liver metastasis, and 129 (50.6%) had lymph node metastasis. There were no significant associations between age; TNM-stage; lymph node metastasis; liver metastasis; or serum levels of AFP. Significant associations were found, however, between gender (P = 0.038 for NLR; 0.012 for MLR), serum levels of CEA (P = 0.003 for NLR; 0.004 for MLR) and CA19-9(P < 0.001 for NLR; P < 0.001 for MLR) (Table 1).

**Table 1:** Clinical characteristics of the patients.

N = 255	Median (IQR) or Mean (std)		Total n (valid percent)	NLR			PLR			MLR		
				<3.13	≥3.13	P	<143.77	≥143.77	P	<0.29	≥0.29	P
Age	63.22 (56,71)	<60	94 (36.9%)	44	50	0.499	50	44	0.203	49	45	0.193
		≥60	161 (63.1%)	67	94		71	90		69	92	
Gender		Male	100 (39.2%)	35	65	0.038	40	60	0.074	36	64	0.012
		Female	155 (60.8%)	76	79		81	74		82	73	
T		1,2	91 (35.7%)	43	48	0.345	43	48	1.000	48	43	0.137
		3,4	164 (64.3%)	64	95		74	85		67	92	
M		0	103 (40.4%)	72	80	0.170	77	75	0.264	76	76	0.186
		1	152 (59.6%)	39	64		44	59		42	61	
TNM-stage		I, II	50 (19.6%)	24	21	0.158	25	20	0.256	27	18	0.055
		III, IV	205 (80.4%)	83	122		92	113		88	117	
CEA	32.00 (110.23)	>10 μg/L	66 (25.9%)	17	49	0.003	28	38	0.325	20	46	0.004
		≤10 μg/L	183 (71.8%)	87	96		88	86		91	83	
AFP	26.80 (176.47)	>25 μg/L	9 (3.5%)	4	5	1.000	4	7	0.540	4	4	1.000
		≤25 μg/L	220 (86.3%)	96	124		109	111		102	118	
CA19-9	303.12 (400.91)	>37 U/ml	135 (52.9%)	43	88	<0.001	50	80	0.006	47	84	<0.001
		≤37 U/ml	106 (41.6%)	61	45		56	41		65	42	
CA125	108.54 (338.96)	>35 μg/L	82 (55.8%)	67	44	0.261	72	49	0.261	69	47	0.261
		≤35 μg/L	65 (44.2%)	80	64		75	59		77	60	
Albumin/globulin ration	1.36 (1.63)	>2	165 (64.7%)	32	58	0.116	39	51	0.405	32	57	0.062
		≤2	90 (35.3%)	75	89		80	84		79	85	
Alkaline phosphatase	220.48 (239.89)	>110	139 (56.3%)	37	102	<0.001	47	92	<0.001	47	91	<0.001
		≤110	108 (43.7%)	69	39		70	38		63	45	
ALT	77.55 (120.19)	>40	118 (47.8%)	38	80	0.001	47	71	0.023	43	74	0.017
		≤40	129 (52.2%)	68	61		70	59		67	62	
AST	83.83 (153.52)	>40	124 (50.2%)	37	87	<0.001	47	77	0.003	43	81	0.001
		≤40	123 (49.8%)	69	54		70	53		67	55	
Total bilirubin	71.09 (117.56)	>17.1	101 (40.2%)	26	75	<0.001	36	65	0.003	30	71	<0.001
		≤17.1	150 (59.8%)	80	70		82	68		81	68	
Gamma-glutamyl transpeptidase	177.81 (245.21)	>40	171 (67.3%)	68	102	0.381	81	89	0.646	73	96	0.835
		≤40	83 (32.7%)	38	45		37	46		37	46	
Neutrophil cell count	6.03 (6.34)	>7.5	56 (22.0%)	3	53	<0.001	16	40	0.002	9	47	<0.001
		≤7.5	198 (78.0%)	104	94		103	95		102	95	
Lymphocyte cell count	1.52 (2.07)	>4	3 (1.2%)	2	1	0.386	3	0	0.063	1	2	0.711
		≤4	251 (98.8%)	105	146		116	135		110	140	
Platelet count	206.03 (99.06)	>300	41 (16.1%)	9	32	0.004	4	37	<0.001	14	27	0.170
		≤300	213 (83.9%)	98	115		115	98		97	115	
Red blood cell count	3.63 (0.69)	>4	81 (31.8%)	35	46	0.811	36	45	0.599	40	40	0.182
		≤4	174 (68.2%)	72	101		83	90		71	102	
Monocyte cell count	1.55 (16.51)	>0.8	31 (12.3%)	4	27	<0.001	11	20	0.169	1	30	<0.001
		≤0.8	222 (87.7%)	102	120		108	114		110	112	
Lymph node metastasis		Yes	129 (50.6%)	55	74	0.869	59	64	0.973	58	71	0.764
		No	126 (49.4%)	56	70		62	70		60	66	
Liver metastasis		Yes	117 (45.9%)	55	66	0.644	49	66	0.201	46	71	0.054
		No	138 (54.1%)	56	78		72	68		72	66	

### Comparison Between Surgical Intervention and Non-Surgical Groups

Two hundred and sixteen patients (84.7%) of the total patients underwent surgery (not including those who had only exploratory laparotomy and/or biopsy), while others chose conservative treatment (conservative treatment refers to non-surgical methods to treat the disease in order to obtain relief of pain, jaundice, and bowel obstruction, and prolongation of life. These methods include chemotherapy, radiation, targeted drugs and Traditional Chinese Medicine). More than a half (163) of these patients received cholecystectomy, of which 70 patients underwent radical cholecystectomy and 11 patients underwent palliative resection. Twenty-eight patients also had their lesions in other location removed, such as liver, omentum majus, mesentery and distant lymph node. Other treatment included percutaneous transhepatic cholangial drainage (PTCD), endoscopic retrograde biliary drainage(ERBD), radiofrequency ablation, transcatheter arterial chemoembolization (TACE) and Iodine particle implantation.

**Figure 1:**
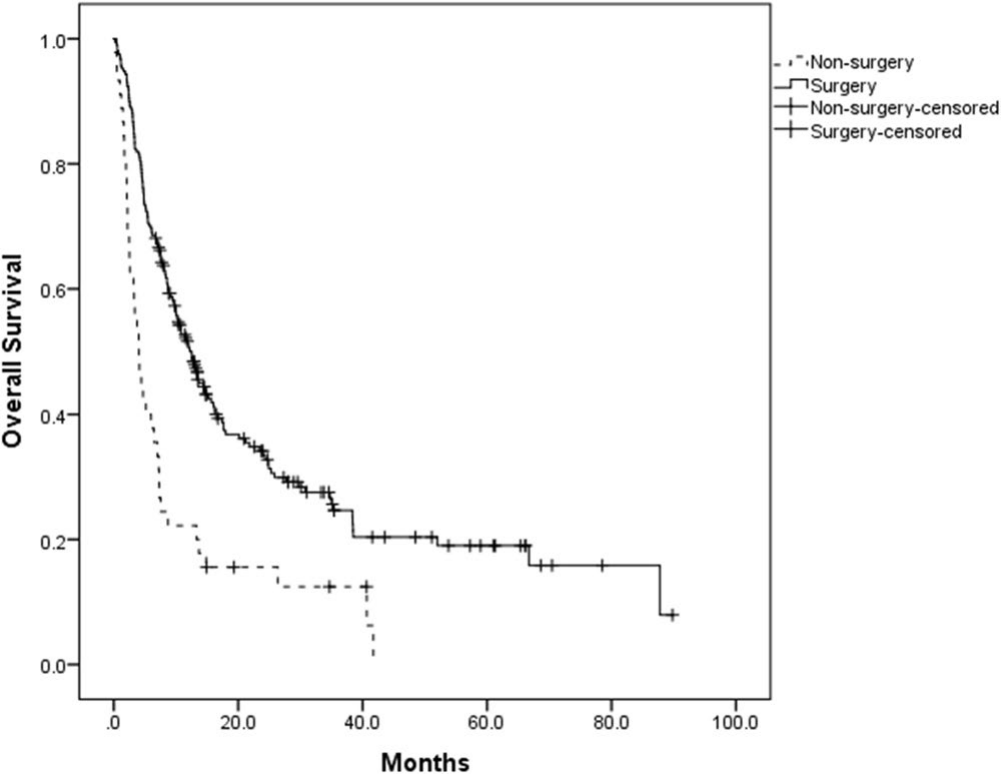
Survival time of non-surgical group and surgical intervention group (39 and 216 patients, respectively).

Among the 255 patients, the longest survival time was 89.8 months and the shortest was 7 days, with the mean survival time being 14.9 months. Non-surgical group had 0.25 as 1-year survival rate, 0.16 as 2-year survival rate and 0.12 as 3-year survival rate, while for surgical intervention group, those numbers were 0.47, 0.31 and 0.24, respectively. Also, surgical intervention group had a 5-year survival rate as 0.19. The median survival time was 4.6 months for non-surgical group, and 12.2 months for surgical intervention group (Fig. 1). Undergoing corresponding surgery had an advantage in survival time (P < 0.001).

Besides, referring to patients in surgery group, those with higher stage (III, IV) tumors show clearly higher hazard ratio than low stage ones (HR 0.278, 95% CI 0.154–0.500, P < 0.001 for TNM-Stage III; HR 0.619, 95% CI 0.428–0.895, P = 0.011 for TNM-Stage IV) in univariate analysis. This was especially noticeable in stage III GBC patients, as it was demonstrated as an independent factor in the influence of staging on surgery (Table 2). Comparison of survival time between non-surgical group and surgical intervention group is shown in Fig. 1.

**Table 2:** Influence of TNM-stage on surgery outcome.

Variable	Parameter	Univariate analysis		Multivariate analysis	
		HR (95% CI)	*p* value	HR (95% CI)	*p* value
TNM stage	I	1.00		1.00	
	II	0.604 (0.322–1.133)	0.116	0.812 (0.349–1.885)	0.627
	III	0.278 (0.154–0.500)	<0.001	0.377 (0.168–0.846)	0.018
	IV	0.619 (0.428–0.895)	0.011	0.778 (0.443–1.364)	0.380

### ROC curve and Prognostic role of NLR, PLR and MLR

**Figure 2:**
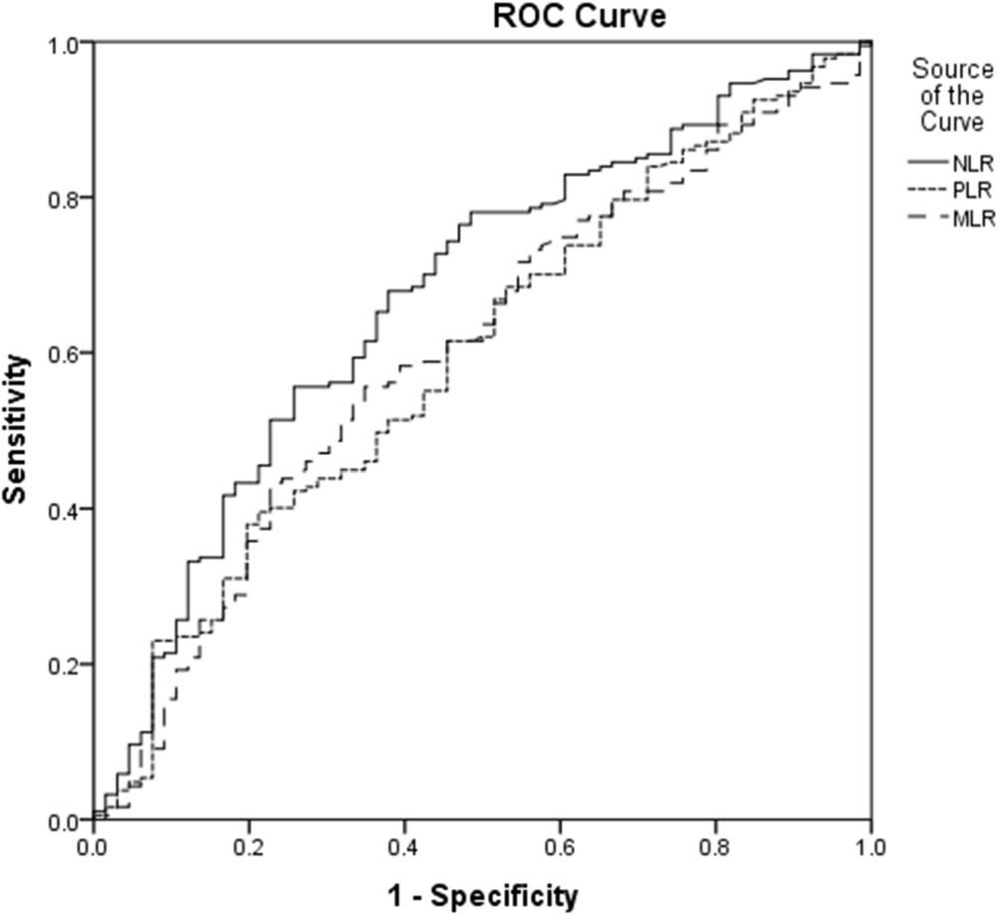
ROC curve analysis for cancer-related survival of NLR, PLR, and MLR (255 patients).

**Figure 3:**
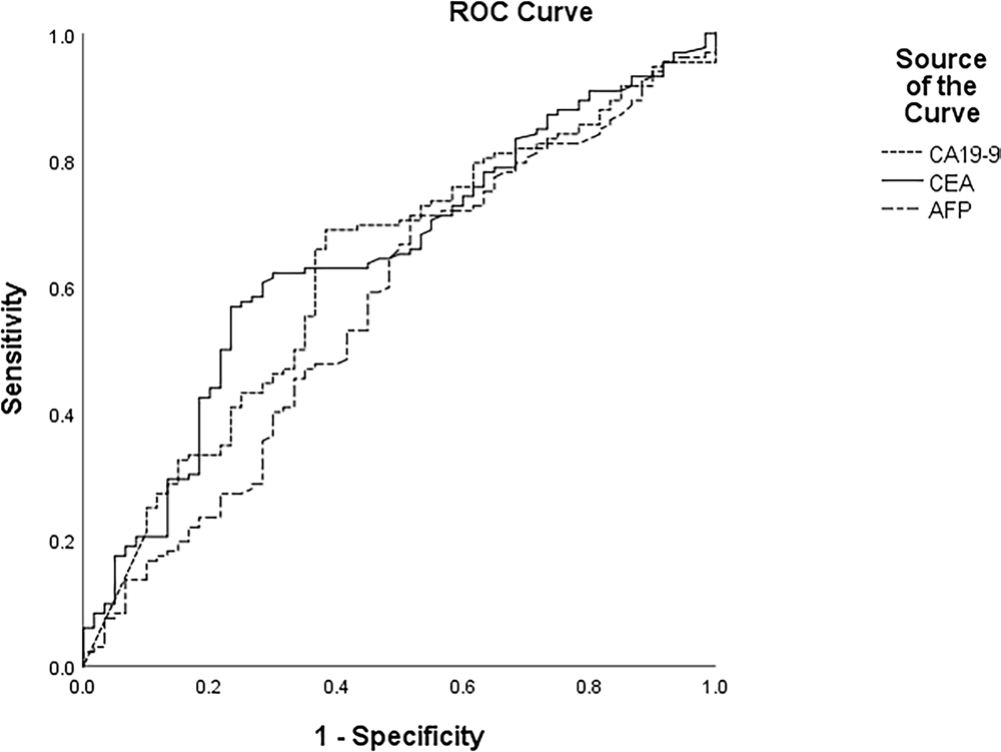
ROC curve analysis for cancer-related survival of CA19-9, CEA and AFP (255 patients).

ROC analysis was applied to calculate the optimal cut off values for NLR, PLR and MLR, and cancer-related deaths being the end point. Figure 2 respectively presented areas under the curve (AUC) of NLR, PLR and MLR were 0.675 (95% CI: 0.600 to 0.751, P < 0.001), 0.599 (95% CI: 0.520 to 0.677, P = 0.017) and 0.607 (95% CI: 0.529 to 0.686, P = 0.009). And we also did ROC analysis on the four more regular tumor markers, the AUCs of CEA, CA19-9, CA125 and AFP were 0.632, 0.634, 0.716 and 0.575, respectively. The AUCs of CA19-9, CEA and AFP being 0.627, 0.646 and 0.569 as shown in Fig. 3. Therefore, all of the three could be used as potential diagnostic biomarkers (AUC > 0.5, P < 0.001, P = 0.017, P = 0.009), and NLR shows higher AUC than the other two. The optimal cut off values for NLR, PLR and MLR were 3.13, 143.77 and 0.29, according to the maximum joint specificity and sensitivity (0.63 and 0.64 for NLR, 0.56 and 0.56 for PLR, 0.59 and 0.59 for MLR).

To rule out some other confounding factors like gender, high-density lipoprotein (HDL) and low-density lipoprotein (LDL), we also did ROC analysis on them too. And the results showed that all of their AUCs were less than 0.50 (0.393 for LDL, 0.456 for HDL and 0.477 for gender).

**Figure 4:**
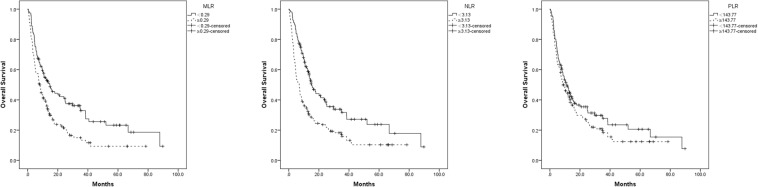
Different survival time between patients with normal and higher NLR (101 and 109 patients, respectively), PLR (104 and 106 patients, respectively), and MLR (105 and 105 patients, respectively) (Cutoffs were evaluated by ROC curve analysis).

The results obtained from the preliminary analysis of the prognostic value of preoperative NLR, PLR and MLR for predicting clinical outcome in GBC patients were shown in Table 3. Univariate analyses showed that high preoperative NLR (≥3.13 versus <3.13; P = 0.039), MLR (≥0.29 versus <0.29; P < 0.001), CEA (>10 μg/L versus ≤10 μg/L; P = 0.006), CA19-9(>37U/ml versus ≤37U/ml; P = 0.001), liver metastasis (P < 0.001), distant metastasis (P < 0.001) were significantly associated with poor overall survival in univariate analysis. Following multivariate analysis illustrated that only liver metastasis was verified as an independent prognostic factor (P = 0.026). We can see patients with these following characteristics: CEA less than 10 μg/L (median OS: 12.02 vs. 8.03 months; P = 0.006); CA19-9 less than 37 U/ml (median OS: 12.90 vs. 8.72 months; P = 0.001); no liver metastasis (median OS: 12.85 vs. 7.77 months; P < 0.001); no distant metastasis (median OS: 12.65 vs. 8.15 months; P < 0.001); NLR less than 3.13 (median OS: 13.00 vs. 8.27 months; P < 0.001); MLR less than 0.29 (median OS: 12.90 vs. 8.73 months; P < 0.001) were associated with better survival (Table 3). Figure 4 showed different survival time between patients with normal and abnormal NLR, PLR and MLR values.

**Table 3:** Univariate and Multivariate analysis of the factors predictive of overall survival in all patients.

Variable	Parameter	Median OS	95% CI	Univariate analysis		Multivariate analysis	
				HR (95% CI)	*p* value	HR (95% CI)	*p* value
Age	<60	11.83	13.54–22.32	1.000	0.357		
	≥60	9.93	13.00–18.48	1.170(0.838–1.633)			
Gender	Male	9.57	11.43–18.97	1.000	0.475		
	Female	11.77	14.31–20.38	1.127(0.812–1.564)			
CEA	>10 μg/L	8.03	8.08–15.25	1.000	0.006	1.000	0.371
	≤10 μg/L	12.02	15.03–20.87	0.298(0.415–0.863)		1.210(0.797–1.838)	
AFP	>25 μg/L	13.80	7.25–24.60	1.000	0.984		
	≤25 μg/L	10.17	13.83–18.88	1.009(0.444–2.291)			
CA19–9	>37 U/ml	8.72	11.30–16.93	1.000	0.001	1.000	0.235
	≤37 U/ml	12.90	15.87–24.50	0.554(0.392–0.784)		1.265(0.858–1.864)	
Lymph node metastasis	No	11.98	15.17–22.52	1.000	0.061		
	Yes	9.85	11.25–17.04	0.859(0.732–1.007)			
Liver metastasis	No	12.85	15.44–21.71	1.000	<0.001	1.000	0.026
	Yes	7.77	10.15–17.22	0.520(0.378–0.716)		0.645(0.438–0.950)	
Distant metastasis	No	12.65	15.99–22.60	1.000	<0.001	1.000	0.080
	Yes	8.15	9.46–15.61	0.515(0.374–0.710)		0.696(0.463–1.045)	
NLR	<3.13	13.00	15.91–23.14	1.000	<0.001	1.000	0.100
	≥3.13	8.27	10.99–17.35	1.945(1.396–2.709)		1.435(0.933–2.207)	
PLR	<143.77	10.80	14.05–21.37	1.000	0.175		
	≥143.77	10.27	12.62–18.99	1.252(0.905–1.733)			
MLR	<0.29	12.90	17.07–24.97	1.000	<0.001	1.000	
	≥0.29	8.73	10.00–15.36	1.836(1.316–2.562)		1.256(0.815–1.934)

### Subgroup analysis

The results obtained from the subgroup analysis were presented in Table 4. In patients with CEA ≤10 μg/L, NLR and MLR were both preoperative predictive with NLR has a higher sensitivity (HR:2.103 vs. 1.918). Also, in patients with CA 19-9 ≤37 U/ml, NLR and MLR were both of predictive importance, with NLR had a higher sensitivity (HR:3.049 vs. 2.430). And in patients with liver metastasis, NLR was the right biomarker to choose, whereas both were right to choose for those without liver metastasis, but NLR had a higher sensitivity (HR: 2.361 vs, 1.998). If patients have discovered distant metastasis already, both biomarkers were right to choose with MLR was more sensitive (HR: 2.048 vs, 1.973). When there were no indications for distant metastasis, then choosing NLR is more realistic (Table 3). Thus, to be more sententious, NLR could be useful in patients with CEA ≤10 μg/L, CA 19-9 ≤37 U/ml, present liver metastasis, distant metastasis or not. Whereas, MLR was applicable in those with CEA ≤10 μg/L, CA 19-9 ≤37 U/ml, liver metastasis or not, and present distant metastasis. And it was noted that, none of the three indexes were statistically significant in non-surgical group, suggesting its application was restricted to patients who would receive surgical treatments.

**Table 4:** Sub-group analyses.

Variables		Surgical group		Non-surgical group	
		HR (95%CI)	p value	HR (95%CI)	p value
**CEA**					
≤10 μg/L	NLR	2.103(1.408–3.140)	<0.001	1.131(0.445–2.878)	0.796
	PLR	1.401(0.939–2.089)	0.099	1.119(0.491–2.552)	0.788
	MLR	1.918(1.274–2.887)	0.002	0.834(0.357–1.949)	0.675
>10 μg/L	NLR	1.163(0.624–2.168)	0.625	1.484(0.318–6.924)	0.616
	PLR	0.834(0.475–1.463)	0.526	1.017(0.304–3.410)	0.978
	MLR	1.264(0.697–2.292)	0.441	1.618(0.435–6.015)	0.473
**CA 19–9**					
≤37U/ml	NLR	3.049(1.752–5.304)	<0.001	0.903(0.293–2.785)	0.859
	PLR	1.436(0.820–2.515)	0.205	1.216(0.402–3.676)	0.729
	MLR	2.430(1.349–4.379)	0.003	1.075(0.350–3.306)	0.900
>37U/ml	NLR	1.188(0.781–1.808)	0.420	1.531(0.444–5.274)	0.500
	PLR	0.895(0.593–1.351)	0.599	0.948(0.396–2.267)	0.904
	MLR	1.213(0.796,1.848)	0.369	0.962(0.387–2.388)	0.933
**Liver metastasis**					
No	NLR	1.581(0.994–2.515)	0.053	0.642(0.202–2.038)	0.452
	PLR	1.163(0.732–1.847)	0.523	0.623(0.186–2.087)	0.443
	MLR	1.637(1.021–2.624)	0.041	0.631(0.195–2.039)	0.631
Yes	NLR	2.361(1.450–3.845)	0.001	1.486(0.556–3.971)	0.429
	PLR	1.337(0.844–2.118)	0.216	1.235(0.535–2.854)	0.621
	MLR	1.998(1.237–3.227)	0.005	1.407(0.566–3.496)	0.463
**Distant metastasis**					
No	NLR	1.770(1.125–2.785)	0.013	0.916(0.331–2.531)	0.916
	PLR	1.107(0.705–1.736)	0.659	0.638(0.228–1.784)	0.392
	MLR	1.562(0.987–2.473)	0.057	0.586(0.210–1.632)	0.586
Yes	NLR	1.973(1.194–3.258)	0.008	1.515(0.506–5.533)	0.457
	PLR	1.327(0.818–2.153)	0.252	1.850(0.712–4.806)	0.207
	MLR	2.048(1.253–3.348)	0.004	2.448(0.717–8.358)	0.153

## Discussion

Surgery is frequently performed on GBC patients, however, there are little studies focused on the exact efficacy of surgical intervention. Also, there is a growing body of evidence suggesting that inflammation plays an important part in numerous cancers such as gastric cancer, esophageal squamous cell carcinoma and non-small cell lung cancer, and these can be used as strong predictors for poor prognosis^10–12^. But its definite influence in GBC is scarcely reported in large cohort study. Therefore, we did this retrospective review of 255 patients with GBC over an 8-year period at West China Hospital of Sichuan University. Our results showed that surgical intervention is, as we hoped, significantly useful in improving the overall survival in our whole patient cohort. And advanced stage tumor patients showed lower survival rate. To our surprise, this phenomenon was more obvious in stage III patients, we assume the reason behind this could be the survival rate in stage IV GBC patients is extremely low, and they are typically believed to be not suitable for surgery. Therefore, we can at least believe that surgery, no matter total and partial resection or palliative operation, is beneficial in improving their life span and is especially good for extending the overall survival of early stage patients. Although surgery is the prime treatment choice for most patients, it is often not easily available as the difficulties in early diagnosis and the poor condition of patients.

Tumor biomarker research has been considered as a research focus for a long time, since uncovering the mystery of the relationship between measurable biological processes and clinical outcomes is significantly useful to expanding our selection of treatments for diseases. Among different types of tumor markers, protein biomarkers, such as IMP2, AFP, B2M, CA15-3, CA19-9, CA-125, cytokeratin and so on, are especially welcomed due to its accessibility.

Up till now, some other bio-markers were found valuable in predicting prognosis of cancer patients, among which NLR, PLR and MLR were closely related to inflammation and immunity status in cancer patients, and they have already been widely applied to estimate the outcomes of patients with various solid tumors. To point out, IMP2 has been found to overexpress in GBC and is significantly correlated with the prognosis^13^. We hope that future study will focus on whether there is an association between IMP2 and inflammatory indexes.

The neutrophils, an essential part of the innate immune system, have an influence on the initiation and progression of tumor in its micro-environment^14^. They show tropism to the original tumor tissue by tumor-secreted chemokines^15^, the ability to prolong life span in cancer cells and facilitate angiogenesis and metastasis of tumor tissues as well as remodel enzymes and pro-angiogenic factors that promote detachment and dissemination of the tumor^16,17^. Plus, it has an impact on organisms’ response reaction to hormones and chemotherapeutic drugs, all indicating that this family of cells has conflicting functions in cancer, although it has always been considered a homogeneous cell population. It has already been elegantly demonstrated that many different types of solid cancer are associated with elevated platelet counts. Numerous animal studies have noticed its contributive effect of platelets upon tumor metastasis^18–20^. The explaining mechanisms refer to the prevention of the circulating tumor cells from the organism’s immune system and supporting extravasation of tumor cells. In addition, monocytes also play an important role in the development of tumor. When they are recruited into the tumor tissue, they will differentiate into tumor-associated macrophages, which support the initiation, local progression and distant metastasis of the tumor tissue^21^. If the NLR level elevated, it means neither the neutrophil amount increases or the lymphocyte amount decreases, which both can be an indication for malignant neoplasms since it shows the disturbance of the inner immune balance inside the organisms. MLR was even recognized as an independent prognostic factor for cancer patients’ overall survival^22^.

Consequently then, we focused on the surgery group of our study and their inflammatory biomarkers as well. What we find suggests that NLR and MLR are two important independent prognostic factors, as patients with high value of these biomarkers are associated with shorter postoperative lifetime. Numerous studies have demonstrated that NLR is a very strong and useful tool, which is consistent with our findings, but the importance and role of MLR is still controversial. At least we have proved its usefulness in the preoperative prognostication of GBC patients.

It is worth mentioning that some of the other studies have come out with different NLR cut-off values from ours, for example 1.94 and 2.3^23,24^, whereas it was 3.13 according to our results. Plus, a relatively large retrospective cohort study also presented their results as the cutoff values of NLR and PLR were 1.94 and 113.34, respectively, with the NLR being the independent prognostic factors as well^25^. Or even, the study of Padrnos on 98 GBC patients showed high NLR’s association with lower hemoglobin and lower albumin, but not prognosis^26^. Taken, our results are mutually corroborated. We assume these differences arise from the sample size and heterogeneity, because these are all single-institution study and their study populations are relatively small. And geographical factors may also play a role in it. It is worthy to mention that another a study conducted in China demonstrates the cut-off value for NLR as 2.16^27^. In our opinion, this study was conducted in Shanxi, a different province of China. According to our research, the epidemiology of GBC in Sichuan and Shanxi is not quite the same. For instance, the composition of gallbladder cancer in all kinds of biliary tract diseases is 1.9 in Sichuan (vs. 3.8 in Shanxi)^28^. Thus, there might be a good reason behind the difference here which calls for a national census for more statistics information.

Our study results, help to the understanding of the nonnegligible role of chronic inflammation in GBC. Another example on the complexity of tumor immunity and chronic inflammation in tumor is a recent study demonstrating that, blocking IL-6 trans-signaling could promote tumor growth, therefore IL-6R inhibitor therapy might not be suitable for GBC or other malignancies associated with bile metabolism^29^. Along with other parallel researches in the future, we hope that the application of immunotherapy on GBC could throw light on the effective treatment of GBC. Our advantages are that we did a relatively large (255 patients) retrospective review and we have a very complete and detailed data base with rather low loss of follow up rate. In addition, we followed up all those people for as long as 5 years for more accurate information. All these factors made this study come up with relatively convincible results. However, we must point out that what we did is a retrospective cohort study. A much more systematic and randomized study would valuable in identifying how surgical intervention interacts with other variables that are believed to be linked to the prognosis of GBC. Also, all our research individuals are Chinese people, clinical differences between races were not reached, so there might be need for more accurate results in other races such as Caucasian. When more documents needed are to increase the dataset, there will be a better understanding of future treatment for gallbladder cancer patients. So, we call for more institutions, to design and carry out perspective studies to confirm our results, which may throw a light on the preoperative evaluation for GBC patients.

### Conclusions

Surgical intervention helps in improving the overall survival in our whole patient cohort, more obviously displayed in early stage GBC patients. The preoperative NLR and MLR were closely associated with the prognosis of GBC patients. These inflammatory factors might be useful for the preoperative prediction of prognosis of patients with GBC. However, further studies are still necessary to investigate the exact mechanisms of NLR and MLR among patients with different stages of gallbladder cancers based on larger sample size.

## Methods

### Patients

A retrospective analysis of patients referred to West China Hospital of Sichuan University with a diagnosis of GBC between 2009 and 2017 was performed. Our study was carried out in conformity to the Declaration of Helsinki (2013) of the World Medical Association. Due to the retrospective nature of the study, informed consent was waived, but our protocols approved by the Ethics Committee of West China Hospital of Sichuan University. We retrospectively recruited all 255 patients who were diagnosed with GBC between 2009 and 2017, all from West China Hospital of Sichuan University.

### Surgical Details

Preoperative examinations included B ultrasound, CT and/or MRI, heart and lung function examination, and laboratory tests. The patient would also have to use PET-CT to take a step to check whether there is a distant transfer. If the examination results showed that tumor can be removed by the operation, and the patient had no obvious surgical contraindications, then he/she would choose intraoperative investigation. After intraoperative investigation shows fair-conditioned lesion site, all of the GBC patients who had decided to undergo the operation would receive radical resections. Otherwise, they could choose whether to have palliative resection or partial resection.

Patients with multiple intrahepatic or distant metastasis and patients with peritoneal planting should be determined either pathological biopsy or palliative resection according to intraoperative exploration situation. The partial hepatectomy was done under the condition of a blood flow controlled system, and the anatomical hepatectomy or non-anatomical hepatectomy was determined by the size and position of the tumor. In the case of the patients who had omentum majus, mesentery or lymph node metastasis, surgeons would perform a corresponding dissection if situation permitted. For patients whose lesions invaded vena cava or portal vein, they were also performed venacavaplasty or portal vein repairment. Solutions like percutaneous transhepatic cholangial drainage (PTCD), endoscopic retrograde biliary drainage (ERBD) were mainly applied to alleviate jaundice. Radiofrequency ablation (RFA), transcatheter arterial chemoembolization (TACE) and Iodine particle implantation were also chosen with regards to the patients’ condition.

### Procedures

Information extracted from clinical history consisted of: (1) demographics (age, sex); (2) clinical stage at presentation, that is, symptoms and signs, lab results, complications, metastatic/localized/locoregional/liver invasion; (3) surgical management; (4) pathological stage classification (according to the 7th TNM staging system of the American Joint Committee on Cancer, AJCC); (5) date of death or last follow-up if date of death unknown. Then we followed up every patient once a month by phone calls, for 5 years from the time of their initial surgery for their survival and prognosis information. The study outcome was cancer-related deaths.

### Statistical Analyses

We perform Chi-square test on categorical variables during univariable analysis and T-test in comparisons between groups of interest. Kaplan-Meier method and the log-rank test were applied to estimate the survival curves and to compare differences in survival between groups. A receiver operating characteristic (ROC) curve was used to pinpoint an optimal cut-off value for inflammatory biomarkers. Cox proportional hazards regression was used to calculate the association of multivariate analyses.

Long term follow-up data were analyzed and reported with overall survival, which was defined as percentage of people in the study group who are alive from date of diagnosis to the last date of contact (September 2017).

We initially conducted analyses for whole cohort of study group (between the surgery group and non-surgery group), concentrating on the overall survival. Then we used subgroup analysis, focusing only on the surgery group by dividing them into two separate cohorts according to their inflammatory biomarkers, in order to investigate the preoperative prognostic value of those biomarkers.

The results are presented as mean ± SEM. P-values < 0.05 were considered statistically significant. All data analyses were performed using SPSS 23.0.
